# Protective Effect of Ethanolic Extract of Grape Pomace against the Adverse Effects of Cypermethrin on Weanling Female Rats

**DOI:** 10.1155/2015/381919

**Published:** 2015-07-22

**Authors:** Abdel-Tawab H. Mossa, Faten M. Ibrahim, Samia M. M. Mohafrash, Doha H. Abou Baker, Souad El Gengaihi

**Affiliations:** ^1^Environmental Toxicology Research Unit (ETRU), Chemical Industries Research Division, Pesticide Chemistry Department, National Research Centre, 33 El Bohouth Street (Former El Tahrir Street), P.O. Box 12622, Dokki, Giza, Egypt; ^2^Pharmaceutical and Drug Industries Division, Medicinal and Aromatic Plants Department, National Research Centre, 33 El Bohouth Street (Former El Tahrir Street), P.O. Box 12622, Dokki, Giza, Egypt

## Abstract

The adverse effect of cypermethrin on the liver and kidney of weanling female rats and the protective effect of ethanolic extract of grape pomace were investigated in the present study. Weanling female rats were given cypermethrin oral at a dose of 25 mg kg^−1^ body weight for 28 consecutive days. An additional two Cyp-trated groups received extract at a dose of 100 and 200 mg kg^−1^ body weight, respectively, throughout the experimental duration. Three groups more served as extract and control groups. Administration of Cyp resulted in a significant increase in serum marker enzymes, for example, aminotransferases (AST and ALT), alkaline phosphatase (ALP), and gamma-glutamyl transferase (GGT), and increases the level of urea nitrogen and creatinine. In contrast, Cyp caused significant decrease in levels of total protein and albumin and caused histopathological alterations in liver and kidneys of female rats. Coadministration of the extract to Cyp-treated female rats restored most of these biochemical parameters to within normal levels especially at high dose of extract. However, extract administration to Cyp-treated rats resulted in overall improvement in liver and kidney damage. This study demonstrated the adverse biohistological effects of Cyp on the liver and kidney of weanling female rats. The grape pomace extract administration prevented the toxic effect of Cyp on the above serum parameters. The present study concludes that grape pomace extract has significant antioxidant and hepatorenal protective activity.

## 1. Introduction 

Pesticides have made valuable contributions to human health by increasing food and fiber production and by reducing the occurrence of vector-borne diseases [1]. Currently, approximately 1,500 active ingredients have been registered as pesticides and formulators mix these compounds with one or more of some 900 inert materials to create approximately 50,000 commercial pesticide preparations registered for use [2]. Roughly, 85% of the pesticides currently used in the world are devoted to the agricultural sector, almost 10% are dedicated to sanitary measures against vectors in public health programs, and the rest are applied in specific sites such as buildings, transport media, and residential areas [3]. In contrast, the use of these toxic materials on man or in his immediate environment has created potential hazards that are of great public health significance [4]. There is a significant concern and focus on the potential increased sensitivity of infants and children to the toxic effects of chemicals [5]. The greater neonatal sensitivity has primarily been attributed to the lack of complete metabolic competence during development [5]. These findings in animals are in agreement with observations in which newborn and young humans have a lower metabolic capacity for CYP450 and PON-1 activity compared to adults [6]. It is noteworthy that most toxicological studies were carried out on adult animals and few data are available concerning the consequences of weanling exposure to pesticides [7].

Synthetic pyrethroids (e.g., cypermethrin) are a diverse class of more than thousand powerful broad-spectrum organic insecticides used in agricultural, domestic, and veterinary applications. However, the toxicity of pyrethroid insecticides to mammals has received much attention in recent years because animals exposed to these insecticides showed changes in their physiological activities besides other pathological features [8, 9]. Due to the lipophilic nature of pyrethroid insecticides [10], they accumulate in biological membranes, leading to stimulation of the production of reactive oxygen species (ROS) and resulting in oxidative damage in mammals [11, 12]. Cypermethrin (RS9-alpha-cyano-3-phenoxybenzyl (1RS)* cis*-trans-3-(2,2-dichlorovinyl)-2,2-dimethyl-cyclopropane carboxylate) has widespread applications in agriculture and household through the world resulting in increased human and their infant's exposure to this compound [13]. It is absorbed through the gastrointestinal and respiratory tracts and confers preferential distribution into lipid-rich internal tissues, including body fat, skin, liver, kidney, ovaries, and the central and peripheral nervous systems [13].

Grapes (*Vitis vinifera*) are the world's largest fruit crop with more than 60 million tons produced annually. About 80% of the total crop is used in wine making [14], and pomace represents approximately 20% of the weight of grapes processed. In Egypt, grapes are considered the second important crop after citrus. The grape growing area is about 152.488 Fadden which produces about 200.000 tons of fruits, in which pomace represents about 10 to 20 thousand tons/year [15]. Grape pomaces (GPs) are characterized by high-phenolic contents because of poor extraction during winemaking, which makes their utilization worthwhile. In recent years, the use of grape seed extracts (GSE) has gained ground as a nutritional supplement in view of its antioxidant activity [16]. The by-products obtained after winery exploitation, either seeds or pomaces, constitute a very cheap source for the extraction of antioxidant flavanones, which can be used as dietary supplements, or the production of phytochemicals, with important medicinal use, in turn providing important economic advantage [17, 18]. Therefore, the current study was designed to evaluate the adverse effects of exposure to Cyp on liver and kidney biomarkers in weanling rats and the ameliorative effect of grape (*Vitis vinifera*) by-products against cypermethrin induced liver and kidney damage.

## 2. Materials and Methods

### 2.1. Chemicals

Cypermethrin (95%) was obtained from Jiangsu Yangnong Chemical Co., Ltd., China. The assay kits used for biochemical measurements of total protein, albumin, urea nitrogen, aspartate aminotransferases (EC 2.6.1.1.), alanine aminotransferases (EC 2.6.1.2), alkaline phosphatase (EC 3.1.3.1), creatinine and gamma-glutamyl transferase (EC 2.3.2.2) in serum were purchased from Bio-Diagnostic Co., Egypt. Kit of total protein was obtained from Stanbio Laboratory, Texas, USA. All other chemicals were of reagent grades and obtained from Sigma-Aldrich and local scientific distributors in Egypt.

### 2.2. Plant Material and Extraction

Thompson seedless (*Vitis vinifera*) pomace was obtained from El Kroom Company, Alexandria, Egypt. One kilogram of grape pomace was dried, ground to a fine powder, and soaked in ethanol (80% v/v) at room temperature for 24 h in the dark. After centrifugation at 4500 rpm for 10 min, the residue was reextracted twice with 80% ethanol as described above. The supernatants were pooled together, concentrated in a rotary evaporator, the dry extract yield being 145.15 g of residue, and then stored at −35°C until being used.

### 2.3. Animals and Care

Weanling female rats weights 50 ± 5 g were obtained from Animal Breeding House of the National Research Centre (NRC), Dokki, Giza, Egypt. Rats were housed in clean plastic cages with free access to food (standard pellet diet) and tap water* ad libitum*, under standardized housing conditions (12 h light/dark cycle, the temperature of 23 ± 2°C, and a minimum relative humidity of 44%) in the laboratory animal room. Animals received humane care, according to the criteria outlined in the “Guide for the Care and Use of Laboratory Animals.”  The Local Ethics Committee at the National Research Centre (NRC), Dokki, Giza, Egypt, approved the experimental protocols and procedures.

### 2.4. Acute Oral Toxicity

The acute oral toxicity was performed according to Economic Cooperation and Development (OECD) 423 guidelines [19]. Five male albino rats (*n* = 5) were used in this study. Rats were fasted overnight with free access to drinking water and then given grape pomace extracts at a dose of 2000 mg/kg and were observed for 24 h and daily for 14 days. After 14 days, no mortality or signs of toxicity were observed. So, 200 mg/kg (1/10th of 2000 mg/kg) was selected as the maximum safety dose with descending dose levels with twofold interval, that is, 100 mg/kg and 200 mg/kg b.wt.

### 2.5. Experimental Design

Female rats were acclimatized for 7 days before treatment and randomly assigned into six groups, seven rats each. Dosages of Cyp and grape pomace extract were freshly prepared and given via oral route for 28 consecutive days. Group I served as control and received corn oil (0.5 mL/rat). Groups II and III received grape pomace extract at doses 100 and 200 mg/kg b.wt., respectively. Group IV was given Cyp at dose 25 mg/kg body weight (1/10 of LD_50_) in corn oil [20]. Groups V and VI were given the same dose of Cyp (25 mg/kg body weight) and the grape pomace extract at doses of 100 and 200 mg/kg b.wt. was given after thirty minutes to groups V and VI, respectively.

### 2.6. Blood Samples

The blood samples were collected, left to clot in clean, dry tubes, and centrifuged at 3000 rpm (600 g) for 10 minutes using Heraeus Labofuge 400R, Kendro Laboratory Products GmbH, Germany, to separate sera. The sera were kept in a deep freezer at −20°C until biochemical markers were analyzed within one week.

### 2.7. Relative Liver and Kidney Weights

The rats were sacrificed by cervical dislocation, and the liver and kidneys were removed and weighed. Then, relative liver and kidney weights were calculated.

### 2.8. Liver and Kidney Dysfunction Biomarkers

All biochemical measurements were determined in serum according to the details given in the kit's instructions and performed by using a Shimadzu UV-VIS Recording 2401 PC (Japan). The activities of cellular enzymes such as AST and ALT were determined according to the methods of Reitman and Frankel [21], ALP according to the method of Young et al. [22], and GGT according to the method of Szasz [23], while the concentrations of total protein, albumin, urea nitrogen, and creatinine were determined according to the methods of Lowry et al. [24], Westgard and Poquette [25], Tietz [26], and Tietz et al. [27], respectively.

### 2.9. Histopathological Examination

After animals were sacrificed, liver and kidneys were removed, washed with normal saline, fixed in 10% formalin, dehydrated in ascending grades of alcohol, and embedded in paraffin wax. Paraffin sections were taken at five *μ*m thickness, stained with haematoxylin and eosin (H&E). The sections were examined for histopathological changes (×160) under light microscope. The liver and kidney fields were scored according to Michael [28] as follows: normal appearance (−), mild (+), moderate (++), severe (+++), and very severe (++++).

### 2.10. Statistical Analysis

Statistical analysis was done using SPSS 17 for windows and the values were expressed as mean ± S.D. The statistical significance of differences between the means was analyzed using one-way analysis of variance (ANOVA) followed by Duncan's test for comparison between different treatment groups. Statistical significance was set at *P* ≤ 0.05.

## 3. Results 

### 3.1. Signs of Toxicity

No mortality occurred during the study period. In contrast, few clinical signs of toxicity such as hyperirritability were noted in Cyp-treated female rats. In addition, there was no effect on food and water consumptions in Cyp-treated female rats compared to control groups (untabulated data).

### 3.2. Body and Relative Organ Weights

**Figure 1:**
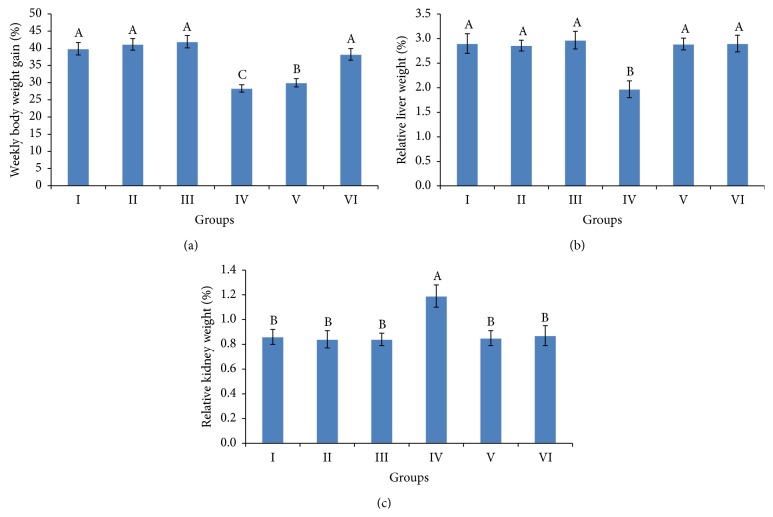
Percent of weekly body weight gain (a) and relative liver (b) and kidney (c) weights of weanling female rats exposed to cypermethrin for 28 days and the protective effect of ethanolic extract of grape pomace (100 and 200 mg/kg b.wt.). The values represented are the means ± S.D. Means having the same letters are not significantly different from each other, *P* < 0.05. Groups: control (I), grape pomace extract at 100 mg/kg b.wt. (II), grape pomace extract at 200 mg/kg b.wt. (III), cypermethrin (IV), cypermethrin and extract at 100 mg/kg b.wt. (V), and cypermethrin and extract at 100 mg/kg b.wt. (VI). Percent of weekly body weight gain = (initial b.wt. − final b.wt./no. of weeks) × 100; relative liver and kidney weight = (liver or kidney weight (g)/final b.wt. (g)) × 100.

Results of weekly body weight gain and relative organ weights of female rats are shown in Figure 1. Results revealed that Cyp-treated female rats induced significant decrease in weekly body weight gain compared to control (Figure 1(a)). Administration of grape pomace extract at 200 mg/kg. b.wt. to Cyp-treated female rats showed insignificant differences in body weight gains, while body weight gain of Cyp-treated rats given 100 mg/kg. b.wt. grape pomace extract was still statistically different from the normal control values. The relative weights of the liver and kidney were significantly changed of Cyp-treated female rats compared to untreated rats (Figures 1(b) and 1(c)). Supplementation of grape pomace extract at 100 and 200 mg/kg. b.wt. to Cyp-treated female rats showed no significant differences in relative weights of liver and kidney as compared to control. Treatment with grape pomace extract at 100 and 200 mg/kg. b.wt. alone did not result in significant change in body and relative organ weights compared to control.

### 3.3. Liver and Kidney Biomarkers

**Figure 2:**
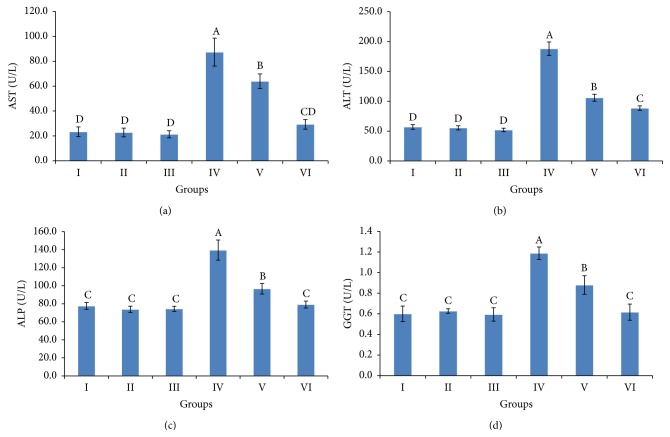
Serum AST (a), ALT (b), ALP (c), and GGT (d) activity of weanling female rats exposed to cypermethrin for 28 days and the protective effect of ethanolic extract of grape pomace (100 and 200 mg/kg b.wt.). The values represented are the means ± S.D. Means having the same letters are not significantly different from each other, *P* ≤ 0.05. Groups: control (I), grape pomace extract at 100 mg/kg b.wt. (II), grape pomace extract at 200 mg/kg b.wt. (III), cypermethrin (IV), cypermethrin and grape pomace extract at 100 mg/kg b.wt. (V), and cypermethrin and grape pomace extract at 200 mg/kg b.wt. (VI). AST, aspartate aminotransferases; ALT, alanine aminotransferases; ALP, alkaline phosphatase; and GGT, gamma-glutamyl transferase.

**Figure 3:**
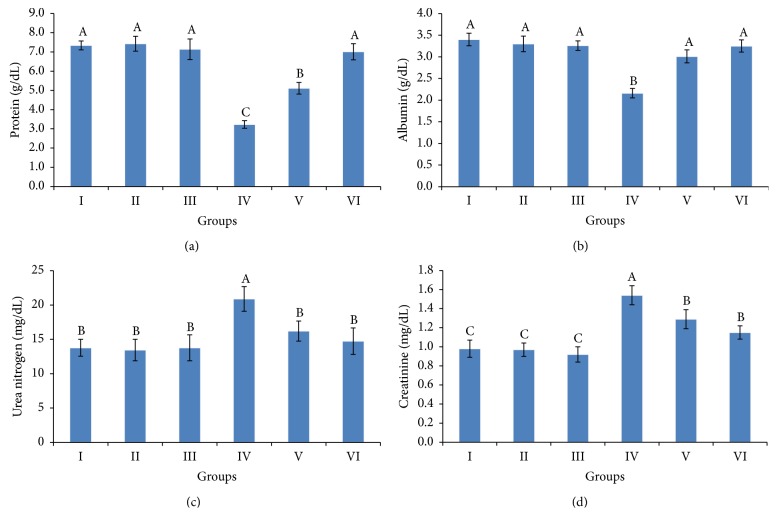
Serum total protein (a), albumin (b), urea nitrogen (c), and creatinine (d) levels of weanling female rats exposed to cypermethrin for 28 days and the protective effect of ethanolic extract of grape pomace (100 and 200 mg/kg b.wt.). The values represented are the means ± S.D. Means having the same letters are not significantly different from each other, *P* ≤ 0.05. Groups: control (I), grape pomace extract at 100 mg/kg b.wt. (II), grape pomace extract at 200 mg/kg b.wt. (III), cypermethrin (IV), cypermethrin and grape pomace extract at 100 mg/kg b.wt. (V), and cypermethrin and grape pomace extract at 200 mg/kg b.wt. (VI).

The results demonstrating the level of liver and kidneys damage sustained following exposure to Cyp are shown in Figures 2 and 3. Significant increases in AST, ALT, ALP, and GGT activities were observed in Cyp-treated rats compared to the corresponding control values (Figure 2). Albumin and total protein levels were significantly decreased in Cyp-treated female rats, while urea nitrogen and creatinine levels showed a significant increase compared to the corresponding control values (Figure 3). However, supplementation of grape pomace extract at 200 mg/kg. b.wt. in conjunction with Cyp reversed the increase in AST, ALP, and GGT activities and urea nitrogen level and the decrease in albumin and total protein levels to within the normal limits. In contrast, ALT activity and creatinine level of Cyp-treated rats given 200 mg/kg. b.wt. grape pomace extract were still statistically different from the normal control values. Coadministration of grape pomace extract at 100 and 200 mg/kg. b.wt. along with Cyp-treated female rats improved liver and kidney functions in a dose-dependent manner. Supplementation of grape pomace extract at 100 and 200 mg/kg. b.wt. to female rats did not result in significant change in serum biomarkers of liver and kidney of female rats.

### 3.4. Histological Studies

**Figure 4:**
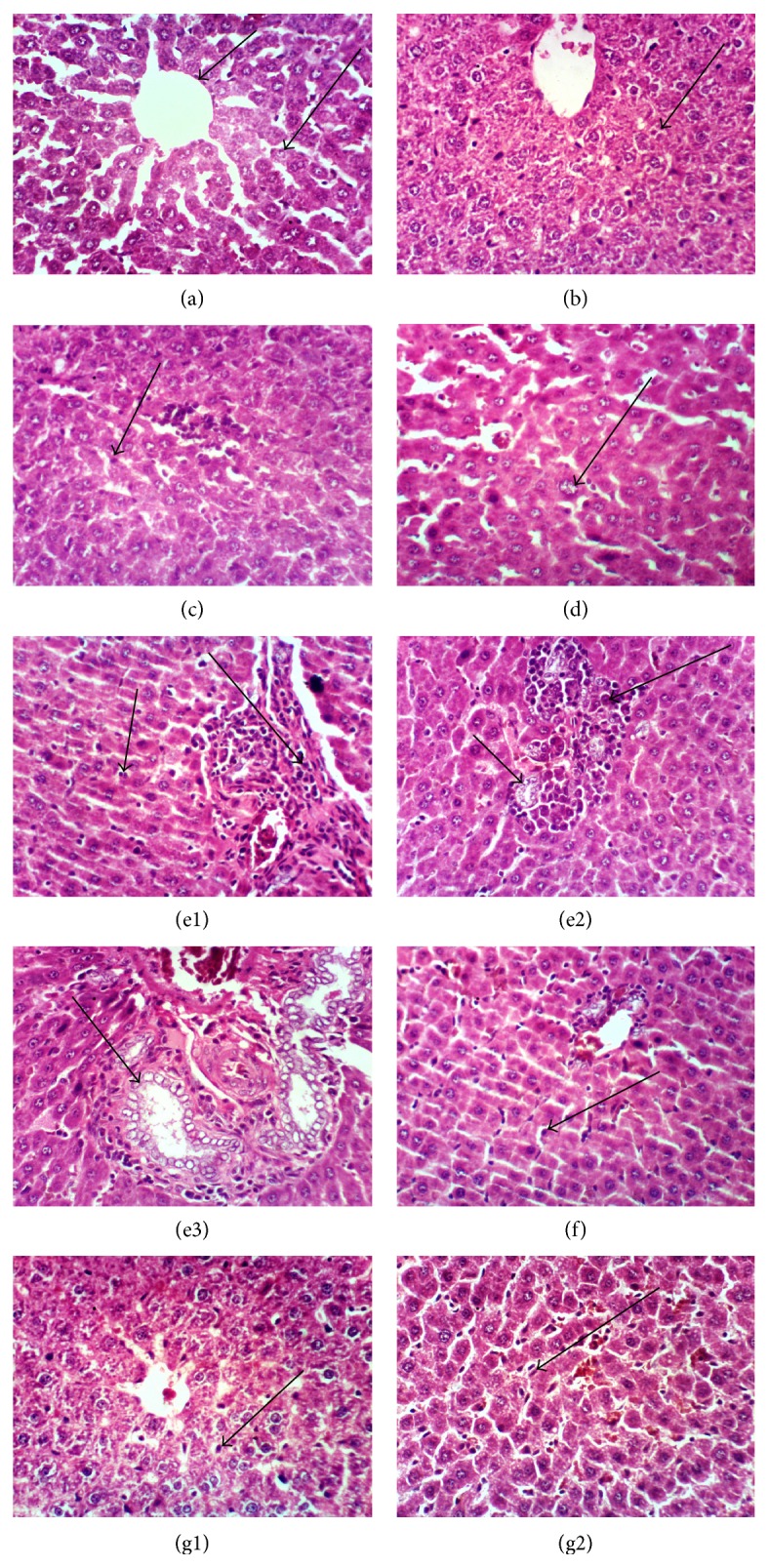
Photomicrograph of liver sections stained by haematoxylin and eosin (H&E) for histological changes (×400). Group 1 showing (a) normal histological structure of hepatic lobule from central vein (small arrow) and hepatic cords (large arrow). Groups II and III (grape pomace extract at 100 and 200 mg/kg. b.wt.) showing slight swelling of hepatocytes (arrow) (b and c). Group IV (cypermethrin at 25 mg/kg b.wt.) showing Kupffer cell activation (arrow) (c), karyomegaly (arrow) (d), Kupffer cells activation (small arrow) and portal infiltration with mononuclear inflammatory cells (large arrow) (e1), dysplasia of bile duct (arrow) and portal infiltration with inflammatory cells (large arrow) (e2), and hyperplasia and hyperactivation of epithelial lining bile duct (arrow) (e3). Group V (grape pomace extract at 100 mg/kg plus cypermethrin) showing Kupffer cell activation (arrow) (f). Group VI (grape pomace extract at 200 mg/kg b.wt. plus cypermethrin) showing slight activation of Kupffer cells (arrow) (g).

**Figure 5:**
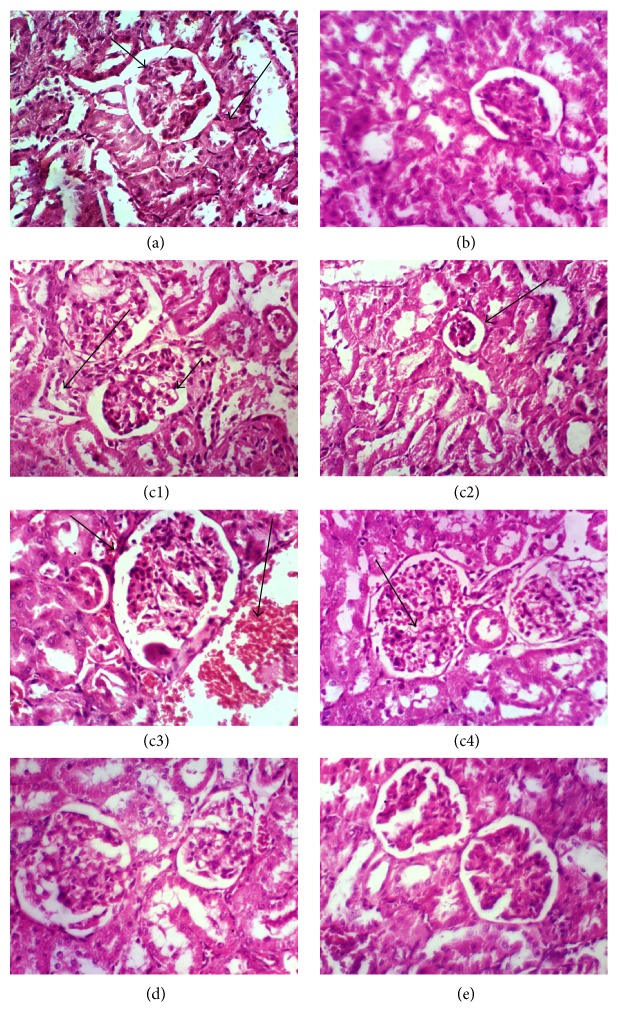
Photomicrograph of kidney sections stained by haematoxylin and eosin (H&E, ×400) for histopathological changes. Group 1 showing (a) the normal histopathological structure of renal parenchyma, normal glomerulus (small arrow), and normal renal tubules (large arrow). Groups II and III (grape pomace extract at 100 and 200 mg/kg. b.wt.) showing no histopathological changes (b). Group IV (cypermethrin at 25 mg/kg. b.wt.) showing vacuolation of endothelial lining glomerular tufts (small arrow) and necrosis of epithelial lining renal tubules (large arrow) (c1), atrophy of glomerular tuft (arrow) (c2), hypertrophy of glomerular tuft (small arrow) and congestion of renal blood vessels (large arrow) (c3), and vacuolation and congestion of glomerular tufts (arrow) (c4). Group V (grape pomace extract at 100 mg/kg plus cypermethrin) showing vacuolation of endothelial lining glomerular tufts (d). Group VI (grape pomace extract at 200 mg/kg b.wt. plus cypermethrin) showing no histopathological changes (e).

As shown in Figures 4 and 5 and Table 1, histological alteration and score of the liver of untreated female rat show normal histological structure of the hepatic lobule (group I). Liver sections of female rats treated with grape pomace extract at 100 and 200 mg/kg. b.wt. showed normal hepatic structure (groups II and III). Cyp treatment (group IV) causes the severe histological changes in the liver, including Kupffer cell, karyomegaly, portal infiltration with mononuclear inflammatory cells, and dysplasia of bile duct, portal infiltration, hyperplasia, and hyperactivation of epithelial lining bile duct surrounded by oval cell proliferation. The liver was almost normal in rats given grape pomace extract at 100 and 200 mg/kg. b.wt. along with Cyp (groups V and VI) with moderate Kupffer cells in a dose-dependent manner of extract (Figure 4).

Light microscopic examination of kidney sections in the control rats (group I) showed the normal histological structure of renal parenchyma (Figure 5). Kidney sections of female rats treated with grape pomace extract at 100 and 200 mg/kg. b.wt. show no histopathological changes and normal renal structure (groups II and III). Cyp treatment (group IV) causes severe vacuolation, necrosis and atrophy of glomerular tuft, hypertrophy, and congestion of renal blood vessels. Administration of grape pomace extract at 100 and 200 mg/kg to Cyp-intoxicated rats shows mild vacuolation of endothelial lining glomerular tufts (group V) and no histopathological changes (group VI).

## 4. Discussion

In the present study, hyperirritability of female rats was recorded in Cyp-treated female rats. The hyperirritability in the treated female rats may be due to the effect of Cyp on axons of the neurons of the peripheral and central nervous system and interacts with the transportation system of sodium ions through the cellular membranes [29]. Sangha et al. [30] found loose fecal pellets and hyperirritability in treated female rats exposed to cypermethrin at 50 mg/kg. b.wt. for 4 weeks.

Results of the present study demonstrate that subchronic Cyp administration produced toxicity in rats as monitored by decrease in body weight gain and increase in relative liver and kidney weights of female rats. In addition, it was observed that there is no effect on feed and water intake in treated female rats. Therefore, the decrease in body weight gain in Cyp-treated female rats may be due to the combined action of neurotoxic effect and oxidative stress. Previous studies show that Cyp caused significant decrease in body weight gain in rats [31] and rabbits [32]. The increase in relative liver and kidney weights could be attributed to the relationship between the liver and kidney weights increases and various toxicological effects or to the reduction in body weight gain of experimental animals [33–35]. Some studies have observed dose-dependent increase in liver and kidney weights of Cyp-treated rats at some acute and subacute dose levels [30, 31, 36, 37]. Coadministration of grape pomace extract to Cyp-intoxicated female rats restored the body weight gains and relative liver and kidney weights to normal weights.

Liver and kidney function biomarkers are a helpful screening tool, which are an effective modality to detect hepatic and renal dysfunction. Our results showed that Cyp caused a significant increase in liver enzymes, that is, AST, ALT, ALP, and GGT in female rats. In fact, aminotransferases are intracellular enzymes, most frequently utilized, and specific indicators of hepatocellular necrosis. AST and ALT catalyze the transfer of the amino acids of aspartate and alanine, respectively, to the keto group of ketoglutaric acid. ALPs are a family of zinc metalloenzymes, with serine at the active center, and they release inorganic phosphate from various organic orthophosphates and are present in nearly all tissues [38]. Also, GGT catalysed the transfer of a glutamyl moiety between peptide donors and amino acid/peptide acceptors [39], involved in the transfer of amino acid across the cell membrane, and had a role in glutathione metabolism, transferring the glutamyl moiety to various acceptor molecules including water, L-amino acids, and peptides [40]. Therefore, the increase in these enzymes in Cyp-treated female rats may be due to liver dysfunction and disturbance in the biosynthesis of these enzymes with alteration in the permeability of the liver membrane takes place [41]. In agreement with our results, a significant increase in liver serum enzymes was observed in male rats given Cyp at doses 12 mg/kg. b.wt. for 30 days [42] and 25 mg/kg. b.wt. for 28 days [43]. Cyp treatment caused severe histological alterations in liver of female rats. These observations indicated marked changes in the overall histoarchitecture of liver in response to Cyp, which could be due to its toxic effects primarily by the generation of ROS [12, 36, 42], causing damage to the various membrane components of the cell. Previous studies reported that Cyp induced histopathological changes in liver tissue of rats [44, 45]. Meanwhile, the changes in liver dysfunction marker enzymes in serum confirmed the histological damage shown in the liver tissue.

In the present study, oral administration of Cyp to weanling female rats caused significant decrease in serum total protein and albumin levels and increase in urea nitrogen and creatinine. The decrease in total protein and albumin in Cyp-treated rats may be due to the liver dysfunctions and disturbance in the biosynthesis of protein. Lakkawar et al. [32] noted that Cyp toxicity decreased level of total protein in serum of young rabbits. In addition, serum levels of urea nitrogen and creatinine are used as indicators of renal function. Therefore, the increase in urea nitrogen and creatinine in Cyp-treated rats may be due to renal failure. The histopathological lesions observed in kidneys of Cyp-treated female rats are in corroboration with the observed biochemical changes. Our results are supported by other studies conducted on Cyp and other insecticides [34, 35, 43, 44].

Supplementation of grape pomace extract at 200 mg/kg. b.wt. in conjunction with Cyp reversed the increase in AST, ALP, and GGT activities, urea nitrogen, and creatinine level and the decrease in albumin and total protein levels to within the normal limits. Therefore, it can be estimated that the recovery of liver and kidney function could be due to the maintenance of cellular integrity in both organs. The protective effect of grape pomace extract may be due to their antioxidant activity, attributed to its major components of phenolic, that is, gallic and cinnamic acid, catechin, rutin, rosmarinic, chlorogenic, caffeic, vanillic, and coumaric acids, and flavonoids [15]. Moreover, the oil of grape seed contains oleic and linoleic acid and *α*-tocopherols [15], responsible for its anti-inflammatory activity [46]. Hence, linoleic acid may also be responsible for reversing the inflammatory features associated with hepatic and renal injury thus adding to the protective effect [47, 48].

## 5. Conclusion 

This study demonstrated the adverse biohistological effects of Cyp on liver and kidney of weanling female rats. Serum marker enzymes such ALT, AST, ALP, GGT, and total protein, albumin, urea nitrogen, creatinine, and histoarchitecture were changed, following Cyp treatment. These results suggested that Cyp has negative effects on hepatorenal cell function. The grape pomace extract administration prevented the toxic effect of Cyp on the above serum parameters. The present study concludes that grape pomace extract has significant antioxidant and hepatorenal protective activity.

## Figures and Tables

**Table 1:** The severity of the reaction in liver and kidney according to the histopathological alteration.

Histopathological alterations	Group					
	I	II	III	IV	V	VI
Liver						
Kupffer cell activation	−	+	+	+++	++	+
Portal infiltration with inflammatory cells	−	−	−	++	−	−
Hyperplasia of bile duct	−	−	−	+++	−	−
Congestion of central vein and hepatic sinusoids	−	−	−	++	−	−
Kidney						
Vacuolization of endothelial lining glomerular tuft	−	−	−	+++	+	−
Vacuolization of epithelial lining renal tubules	−	−	−	++	−	−
Necrosis of epithelial	−	−	−	++	−	−

Nil (−), mild (+), moderate (++), and severe (+++). Groups: control (I), grape pomace extract at 100 mg/kg b.wt. (II), grape pomace extract at 200 mg/kg b.wt. (III), cypermethrin (IV), cypermethrin and grape pomace extract at 100 mg/kg b.wt. (V), and cypermethrin and grape pomace extract at 100 mg/kg b.wt. (VI).
